# How nurses’ job characteristics affect their self-assessed work environment in hospitals— Slovenian use of the practice environment scale of the nursing work index

**DOI:** 10.1186/s12912-023-01261-5

**Published:** 2023-04-06

**Authors:** Brigita SKELA-SAVIČ, Walter SERMEUS, Simon DELLO, Allison SQUIRES, Mateja BAHUN, Bojana LOBE

**Affiliations:** 1 Angela Boškin Faculty of Health Care, Spodnji Plavž 3, Jesenice, 4270 Slovenia; 2 Leuven Institute for Healthcare Policy, Department of Public Health & Primary Care, Katholieke Universiteit Leuven, Kapucijnenvoer 35/4, Leuven, 3000 Belgium; 3 Dept. of General Internal Medicine, Grossman School of Medicine, Meyers College of Nursing, New York University, New York, USA; 4 Faculty of Social Sciences, University of Ljubljana, Kardeljeva ploščad 5, Ljubljana, 1000 Slovenia

**Keywords:** PES-NWI scale, Instrument validation, Job characteristics, Nurse involvement, Nurse-Physician relationship, Advancement opportunities, Educational opportunities, Staff development

## Abstract

**Background:**

Nurses’ work environment influences nursing practice. Inappropriate working conditions are the result of underdeveloped workplace infrastructure, poor work organisation, inadequate education, and inappropriate staffing norms. The aim of this study was to describe and examine the predictors that affect nurses’ work environment using the Practice Environment Scale of the Nursing Work Index (PES-NWI).

**Methods:**

The validation of the PES-NWI was made. Nurse-reported job characteristics were used as independent variables. The sample included 1,010 nurses from adult surgical and medical units at 10 Slovenian hospitals. The Nurse Forecasting (RN4CAST) protocol was used. Permission to conduct the study was obtained from the National Medical Ethics Committee.

**Results:**

The PES-NWI mean (2.64) was low, as were job and career satisfaction at 2.96 and 2.89, respectively. The PES-NWI can be explained in 48% with ‘Opportunities for advancement’, ‘Educational opportunities’, ‘Satisfaction with current job’, ‘Professional status’, ‘Study leave’, and ‘Level of education’. A three-factor solution of PES-NWI yielded eight distinct variables.

**Conclusions:**

The obtained average on the Nursing Work Index was one of the lowest among previously conducted surveys. Nurses should be recognized as equals in the healthcare workforce who need to be empowered to develop the profession and have career development opportunities. Inter-professional relations and equal involvement of nurses in hospital affairs are also very important.

**Trial registration:**

This is a non-intervention study – retrospectively registered.

## Background

Recent studies have revealed that nurses’ work environments play an important role in their ability to provide quality care [1–5]. Despite national scientific evidence, nurses in Slovenia continue to face unsafe work conditions, understaffing, inappropriate nursing staff structure in terms of educational background, low decision authority, and high job demands [6, 7]. This has had a major impact on the professionalization of nursing and its development as a science [8, 9].

In the literature, nurses’ experiences of working in an unhealthy environment have been mentioned as an important reason for a decrease in the nursing workforce and have been found to adversely affect both nurse and patient outcomes [3, 10, 11]. Negative nursing outcomes related to an inappropriate work environment include exhaustion, turnover, and job dissatisfaction [12]. Nurses in more favourable work environments had 28–32% lower odds of developing job dissatisfaction or burnout and to have an intention to leave, while patients had 8% lower odds of experiencing an adverse event or even death [3]. According to research evidence, paying attention to variables such as work environment and organisational characteristics is crucial for improving the work environment and increasing nurses’ intention to stay [4]. Thus, nurses’ work environment should be considered a global critical predictor influencing quality of care [13].

Important predictors for job satisfaction/dissatisfaction include nursing leadership, response/teamwork, and resourcing; these are especially important to provide high-quality care in the nursing environment [14]. Autonomy in decision-making and the possibilities of career-long professional development are becoming key factors for prospective nurses and for the development of young nurses [7]. Nurses seem to be more likely to stay in their job if crucial work environment factors such as effective leadership, empowerment possibilities, career development opportunities, and a positive organisation climate are met [4].

Inter-professional collaboration is also an important part of the work environment. Poor nurse-physician collaboration appears to be a crucial factor accounting for nurses’ distress [15], while good nurse-physician collaboration can improve patient outcomes [16, 17]. Also, ineffective or non-existent collaborative interaction leads to decreased empowerment, increased burnout, decreased job satisfaction, and increased turnover among nurses [15, 18].

Much is already known about the work environment of nurses, but its importance is still under-recognised by leaders. Nurse leaders can play an important role in establishing better work environments for nurses by adjusting process improvements that require active involvement of frontline nurses and nurse executives [19].

Those in charge of nursing like to assume that a pay rise will solve all nurses’ problems, but research shows that this is not the case. Wage is important for good nurse outcomes, but it does not diminish the significant influence of the work environment [20]. Different identifying factors are stronger than salary and influence the positive environment. Al Sabei et al. [21] defined positive work environment factors as autonomy, environmental control, the relationship between doctors and nurses, and organisational support. Poor practice environments were found to have negative consequences not only on nurses but also on patients [1, 22]. Nurse managers need to build a supportive work environment as an effective way to increase nurses’ psychological bonding and enhance positive work outcomes that may in turn enhance organisational performance and their work engagement [19]. Findings of the studies reviewed by Wei et al. [2] indicated that nurse managers’ leadership ability was significantly positively associated with nurses’ perceptions of the work environments.

Nursing work environments have been measured with various instruments and new instruments are still developed today [23], mainly in hospital settings [24]. A recent meta-analysis reported that the Practice Environment Scale of the Nursing Work Index (PES-NWI) [25] and the individual subscales were a reliable indicator of the professional practice environment [26]. The literature review conducted by Swiger et al. [1] revealed a significant association between the nursing outcomes of interest in at least one of the PES-NWI subscales and/or the composite score.

## Methods

### Aim

The aim of this study was to describe and examine the predictors that affect nurses’ work environment using the Practice Environment Scale of the Nursing Work Index (PES-NWI). The sub-objectives of this study were to describe and examine (1) the country-specific context of nurses’ work environment in Slovenian hospitals using the scale PES-NWI; (2) the differences between two professional groups involved in nursing in Slovenia: registered Nurses (RNs) and Health Care Assistants (HCAs); and (3) predictors that can predict nurses’ self-assessment of their work environment and that can be used by managers to improve nurses’ working conditions.

### Design

A cross-sectional explorative research design was employed. The same research protocol and measures employed in RN4CAST were used also to collect data in this study [27]. STROBE checklist was used to report the cross-sectional study.

### Instrument

The PES-NWI [25] scale was used. The scale has 32 positively phrased items, each with four possible answers (1 – Strongly Disagree, 2 – Somewhat Disagree, 3 – Somewhat Agree, 4 – Strongly Agree) and five subscales: ‘Nurse participation in hospital affairs’ (8 items); ‘Nursing foundations for quality of care’ (9 items); ‘Nurse manager ability, leadership and support of nurses’ (4 items); ‘Staffing and resource adequacy’ (4 items); and ‘Collegial nurse-physician relationships’ (7 items). Forward and backward translations were conducted by two translators specializing in healthcare terminology. The same translation methodology (a forward and backward translation with two translators) used for the RN4CAST study [28] was applied.

In addition to standard demographic data (gender, age, education, length of employment, full-time work), self-assessment of nurses’ job characteristics data was also collected with two scales: satisfaction with current job and nursing as a career, different aspects of the job (work schedule flexibility, opportunities for advancement, independence at work, professional status, wages, educational opportunities, annual leave, sick leave, study leave), (scale: 1 – Very Dissatisfied, 2 – A Little Dissatisfied, 3 – Moderately Satisfied, 4 – Very Satisfied); and conditions in the work environment (1 – Poor, 2 – Fair, 3 – Good, 4 – Excellent), also used in the RN4CAST instrument [27].

### Settings and sample characteristics

Per the RN4CAST protocol [27], all Slovenian hospitals with adult surgical and medical units were invited to participate in the study (N = 12). Of these, ten hospitals (n = 10) confirmed their participation. Participating hospitals included eight general hospitals and two larger hospitals that provide also tertiary services (rare diseases, complex medical treatments) in addition to general hospital services. Specialized units (e.g. intensive care, high dependency units, transplant care units, paediatric units, geriatric units, and long-term care nursing units) were excluded from the sampling frame.

All employed nurses (RNs) and health care assistants (HCAs) at the included units (N = 2,813) who were providing direct nursing care to patients were invited to participate. Nurses who were on sick leave, maternity leave, or on vacation were excluded. Slovenia faces a major shortage of RNs, with only 30% of RNs in the nursing workforce group, a far cry from the recommended 80% of nurses with a Bachelor of Science in Nursing degree (BSN) [29, 30]. RNs are defined as those meeting the European Union definition of trained and licensed nurses according to Directive 2005/36/EC [31]. In Slovenia, this means a professional bachelor’s degree in nursing, level six of the European Qualifications Framework (EQF). The educational background of HCAs is four years of secondary school for healthcare technicians, level four of the EQF.

### Data collection process

The data collection process started in February 2020. The majority of data was collected between February 10 and March 7, 2020, before the onset of the COVID-19 pandemic. Only one hospital collected data from 8 to 20 June, 2020, when Slovenian hospitals again functioned normally—the first wave of the epidemic in Slovenia was weak, and the epidemic ended on May 31, 2020. The resulting sample of data after the onset of the epidemic represents less than 10% of the total sample. A cross-sectional approach was used; each hospital had two weeks for data collection. A paper-and-pencil approach was used, printed questionnaires were distributed by management-designated research coordinators. Respondents returned them in a sealed envelope at the agreed collection point.

### Ethical approval

Permission to conduct the study in Slovenian hospitals was obtained from the National Medical Ethics Committee (No. 0120–488/2019/6, January 7, 2020). This is not an experimental study, it is a non-experimental explorative study. All methods were carried out in accordance with the relevant guidelines and regulations: we followed the Guidelines for Research Ethics in the Social Sciences and the Humanities [32], and the Helsinki Declaration [33]. Each hospital as a legal guardian confirmed its participation by adopting a decision at the relevant expert board or ethical committee. Participants received written information about different aspects of the study; their rights on voluntary participation and withdrawal from the study at any time were explained to them as well as their privacy and confidentiality rights. The participants also gave their written informed consent to participate in the study and permission to use the data collected at the national level for professional and scientific purposes. Informed consent to publish in scientific journals (online, open-access) was also obtained from the study participants.

### Data analyses

Data were analysed with statistical software SPSS 22. Basic univariate and bivariate statistical analysis were conducted, including descriptive statistics and multivariant analyses. A mean composite score of each PES-NWI subscale and an overall composite score were calculated. Lake [25] considers 2.5 to be the neutral midpoint for a four-point response set, with values above 2.5 indicating agreement and a favourable environment and below 2.5 disagreement or an unfavourable environment. Cronbach’s alpha was used to check the reliabilities of the measured scales. Further, factor analysis was performed to investigate the construct validity of the measured scales. First, the confirmatory factor analysis (CFA) and principal component analysis (PCA) were used, followed by a principal axis factor analysis (PAF) with oblique rotation (Oblimin) to achieve the optimal coherent construct of the instrument. The lower limit of communalities was set at 0.400. The method of rotation with Keiser normalization was used since correlations between factors were observed. We conducted Bartlett’s sphericity test (p < 0.05) and the KMO test (> 0.6), which showed that the sample was of a suitable size [34]. The linear regression model was used. Statistical significance was measured at the p < 0.05 level.

## Results

### Participants

The population of nursing staff working in adult surgical and medical units in the participating hospitals ranged from 49 to 1,197, including smaller and regional hospitals and two larger hospitals in the two largest Slovenian cities (Ljubljana and Maribor). The response rate was 35.91% (n = 1,010), hospital response rates ranged from 23.1 to 61.2%. The lowest and highest number of responses per hospital were 42 and 281, respectively. Respondents included 848 (83.96%) females and 160 (15.84%) males. The sample included 403 RNs with a bachelor’s degree (40%) and 605 (60%) HCAs. The average length of employment of respondents was 21.42 years (SD = 3.40), and employment in nursing was 15.34 years (SD = 11.12). Their average age was 37.02 (SD = 10.65) years. Almost all (96%, n = 970) were employed full time.

### Reliability and construct validity of PES-NWI scale

The reliability test of PES-NWI was very good (n = 1,008, α = 0.937), but not all individual subscales proved to be as highly reliable. First, a confirmatory factor analysis (CFA) was carried out on the existing five subscales/factors structure. The reliability was as follows: ‘Staffing and resource adequacy’, α = 0.636; ‘Nurse manager ability, leadership and support of nurses’, α = 0.727; ‘Nursing foundations for quality of care’, α = 0.818; ‘Nurse participation in hospital affairs’, α = 0.843; and ‘Collegial nurse-physician relationships’, α = 0.894.

Second, factor analysis was used to identify a set of coherent subscales in our sample. The first PCA yielded six factors and explained 57.13% of the variance. Secondly, the PAF method required the elimination of eleven statements with communalities < 0.400 (see the unweighted results in the column Extraction Communalities, Table 1).

**Table 1:** Mean values and extraction of communalities—Principal Axis Factorial analysis

Statements:	*n*	M	SD	Extraction Communalities
1. Adequate support services allow me to spend time with my patients.	1005	2.55	0.892	0.202
2. Physicians and nurses have good working relationships.	1003	2.85	0.751	**0.487**
3. A supervisory staff that is supportive of nurses.	999	2.96	0.765	**0.441**
4. Active staff development or continuing education programs for nurses.	993	2.77	0.829	**0.633**
5. Career development/clinical ladder opportunity.	1004	2.60	0.889	**0.740**
6. Opportunity for registered nurses to participate in policy decisions.	967	2.64	0.877	**0.459**
7. Physicians value nurses’ observations and judgments.	1007	2.59	0.833	**0.558**
8. Enough time and opportunity to discuss patient care problems with other nurses.	1008	2.56	0.831	0.288
9. Enough registered nurses on staff to provide quality patient care.	994	2.08	0.980	0.383
10. A nurse manager who is a good manager and leader.	985	3.34	0.774	0.**537**
11. A chief nursing officer who is highly visible and accessible to staff.	997	2.77	0.926	0.362
12. Enough staff to get the work done.	994	1.90	0.924	**0.650**
13. Physicians recognize nurses’ contributions to patient care.	1003	2.44	0.841	**0.570**
14. Praise and recognition for a job well done.	1004	2.41	0.889	0.396
15. High standards of nursing care are expected by the management.	998	2.88	0.838	0.307
16. A chief nursing officer is equal in power and authority to other top-level hospital executives.	989	2.62	0.885	**0.458**
17. A lot of team work between nurses and physicians.	1006	2.74	0.866	**0.549**
18. Opportunities for advancement.	1003	2.33	0.860	**0.473**
19. A clear philosophy of nursing that pervades the patient care environment.	996	2.63	0.795	**0.494**
20. Working with nurses who are clinically competent.	995	3.21	0.746	0.297
21. Physicians respect nurses as professionals.	1003	2.45	0.877	**0.565**
22. A nurse manager who backs up the nursing staff in decision making, even if the conflict is with a physician.	997	3.17	0.862	**0.519**
23. Management that listens and responds to employee concerns.	1000	2.58	0.866	**0.474**
24. An active quality assurance program.	994	2.59	0.821	**0.505**
25. Registered nurses are involved in the internal governance of the hospital (e.g., practice and policy committees).	976	2.72	0.846	**0.555**
26. Collaboration between nurses and physicians.	999	2.91	0.783	**0.571**
27. A preceptor program for newly hired nurses.	994	2.76	0.934	0.340
28. Nursing care is based on a nursing rather than a medical model.	978	2.68	0.759	0.389
29. Registered nurses have the opportunity to serve on hospital and nursing committees.	963	2.69	0.844	**0.527**
30. Physicians hold nurses in high esteem.	996	2.15	0.863	**0.581**
31. Written, up-to-date care plans for all patients.	990	2.76	0.903	0.254
32. Patient care assignments that foster continuity of care (i.e., the same nurse cares for the patient from one day to the next).	1001	2.15	0.944	0.191
Together (min. = 1.03; max = 3.94)		2.64	0.491	

Note. *n* – Number of answers, M – Mean on a 4-point scale (1 – Strongly Disagree, 2 – Somewhat Disagree, 3 - Somewhat Agree, 4 – Strongly Agree), SD – Standard deviation, *p = P-value*

We found that three factors had only two statements which did not constitute a good coherent construct. Therefore, a three-factor solution was adopted. PAF was performed again with the remaining statements, two of those had communalities < 0.400 (i.e. items 12, 25). We excluded them; the total scale had a Cronbach’s alpha of 0.917. The second PAF with a scree plot yielded a three-factor solution (Table 2). Variance was explained in 50.37% of cases. All communalities were > 4.00. We used Oblimin rotation with Kaiser normalisation (KMO = 0.930; Bartlett p < 0.001). The first factor included nine statements explaining 37.86% of the variance and was named ‘Equal development of nurses and their participation in hospital affairs’. The second factor included seven statements that explained the variance in 7.26% of cases, the factor had negative weights and was named ‘Collegial nurse-physician relationships’. The third factor included three statements explaining 5.25% of the variance. We named it ‘Nurse manager ability, leadership and support of nurses’ (Table 2).

**Table 2:** Results of Principal Axis Factorial analysis with content of three factors

	Factors			
	1	2	3	
**F 1 - Equal development of nurses and their participation in hospital affairs (α = 0.884; 37.86% of variance)**				
25. Registered nurses are involved in the internal governance of the hospital (e.g. practice and policy committees).	**0.781**	-0.011	-0.089	
29. Registered nurses have the opportunity to serve on hospital and nursing committees.	**0.748**	0.023	-0.098	
6. Opportunity for registered nurses to participate in policy decisions.	**0.719**	0.012	-0.041	
19. A clear philosophy of nursing that pervades the patient care environment.	**0.477**	-0.165	0.207	
5. Career development/clinical ladder opportunity.	**0.460**	-0.081	0.280	
24. An active quality assurance program.	**0.444**	-0.215	0.148	
18. Opportunities for advancement.	**0.440**	-0.187	0.179	
16. A chief nursing officer is equal in power and authority to other top-level hospital executives.	**0.438**	-0.119	0.208	
4. Active staff development or continuing education programs for nurses.	**0.430**	0.009	0.350	
** F 2 – Collegial nurse-physician relationships (α = 0.894; 7.26% of variance)**				
7. Physicians value nurses’ observations and judgments.	0.010	**-0.771**	-0.072	
30. Physicians hold nurses in high esteem.	0.085	**-0.745**	-0.080	
13. Physicians recognize nurses’ contributions to patient care.	0.068	**-0.743**	-0.068	
21. Physicians respect nurses as professionals.	0.002	**-0.743**	0.015	
2. Physicians and nurses have good working relationships.	-0.149	**-0.736**	0.094	
26. Collaboration between nurses and physicians.	0.064	**-0.691**	0.041	
17. A lot of team work between nurses and physicians.	0.085	**-0.660**	0.056	
** F 3 - Nurse manager ability, leadership and support of nurses (α = 0.751; 5.25% of variance)**				
10. A nurse manager who is a good manager and leader.	0.016	0.090	**0.774**	
22. A nurse manager who backs up the nursing staff in decision making, even if the conflict is with a physician.	0.039	-0.011	**0.635**	
3. A supervisory staff that is supportive of nurses.	-0.056	-0.239	**0.574**	

F1 – Equal involvement of nurses and their participation in hospital affairs, F2 – Collegial nurse-physician relationships, F3 - Nurse manager ability, leadership and support of nurses

We compared the reliability of the recommended five subscales of Lake [25] and the three obtained factors. The majority of items in the subscale ‘Nursing foundations for quality of care’ have communalities < 0.400; no factor with such content was obtained. The same goes for ‘Staffing and resource adequacy’. Considering the items that were ranked in Factor 1 and had communalities < 0.400, we found that three subscales of Lake [25] were substantively ranked in Factor 1 (‘Equal development of nurses and their participation in hospital affairs’), with a total of nine statements, while the subscales ‘Collegial nurse-physician relationships’ and ‘Nurse manager ability, leadership and support of nurses’ were substantively fully comparable to new factors 2 and 3.

### Explorative results

The mean value for nurses’ current work environment (PES-NWI) was 2.65 (SD = 0.49) (Table 1), no differences between the two occupational groups were established (Table 3). The average levels of satisfaction with the current job, career, and work environment are shown in Table 3. The overall average of the different job aspects had a mean of 2.73 (SD = 0.66), with differences between the two occupational groups (p < 0.001). Table 3 reveals the self-assessment results for the nine aspects of the job in more detail. The lowest was wage, followed by opportunities for advancement. Differences are shown according to educational qualifications (Table 3).

**Table 3:** Reported job characteristics, differences BSN/HCA and explorative analysis between variables

*Variables*	*n*	M (SD)	HCA/BSN p	F1	F2	F3
Satisfaction – current job	1,003	2.96 (0.733)	**0.002**	0.411^**^	-0.424^**^	0.464^**^
Satisfaction – career	988	2.89 (0.791)	**<0.001**	0.269^**^	-0.220^**^	0.292^**^
Work environment rate (PES-NWI)	998	2.65 (0.750)	0.682	0.430^**^	-0.423^**^	0.502^**^
*Aspects of job*						
1-Work schedule flexibility	1,000	2.83 (0.910)	**0.002**	0.361^**^	-0.302^**^	0.415^**^
2-Opportunities for advancement	1,000	2.55 (0.958)	**<0.001**	0.542^**^	-0.397^**^	0.455^**^
3-Independence at work	995	2.98 (0.792)	**0.002**	0.421^**^	-0.355^**^	0.445^**^
4-Professional status	1,003	2.79 (0.947)	**0.009**	0.438^**^	-0.434^**^	0.432^**^
5-Wages	1,002	2.18 (0.952)	**<0.001**	0.358^**^	-0.308^**^	0.293^**^
6-Educational opportunities	1,004	2.69 (0.898)	**<0.001**	0.524^**^	-0.372^**^	0.481^**^
7-Annual leave	1,001	2.89 (0.889)	**<0.001**	0.369^**^	-0.295^**^	0.367^**^
8-Sick leave	936	2.99 (0.896)	0.052	0.349^**^	-0.308^**^	0.353^**^
9-Study leave	829	2.75 (1.031)	0.122	0.423^**^	-0.321^**^	0.358^**^
*Factors of PES-NWI*						
F1 - Equal involvement of nurses and their participation in hospital affairs	861	2.60 (0.85)	0.528	1		
F2 - Collegial nurse-physician relationships	861	2.59 (0.831)	0.792	-0.618^**^	1	
F3 - Nurse manager ability, leadership and support of nurses	861	3.16 (0.80)	0.081	0.523^**^	-0.539^**^	1

Note. *n* – Number of answers, M – Mean (4-point scale), SD – Standard deviation, *p = P-value*

**Correlation is significant at the 0.01 level (2-tailed)

According to explorative analysis, no correlation was shown between gender, educational background, age, and years of employment in nursing and the mean value of the three factors. A significant correlation was found to exist between the three factors and job characteristics. Also, the developed factors were in significant correlation (Table 3). The majority of correlations could be classified as medium (r = 0.300 to 0.600) [34].

Table 4 shows two linear regression model results. In the first, Regression Model 1, the PES-NWI scale with all 32 statements can be explained in 48%. The most important explaining variables are: ‘Opportunities for advancement’, ‘Satisfaction with current job’, ‘Educational opportunities’, and ‘Professional status’. Weaker explaining variables include ‘Study leave’ and ‘Level of education’ (Table 4).

In the second Regression Model, Factor 1 (‘Equal involvement of nurses and participation in hospital affairs’) was explained in 40.4% by beta size variables: ‘Opportunities for advancement’ and ‘Educational opportunities’, followed by ‘Study leave’ and ‘Level of education’. Factor 2 (‘Collegial nurse-physician relationships’) was explained in 26.8% by the following beta-size variables: ‘Satisfaction with current job’, ‘Professional status’, ‘Opportunities for advancement’, and ‘Educational opportunities’. Factor 3 (‘Nurse manager ability, leadership and support of nurses’) was explained in 35.4% by beta-size variables ‘Educational opportunities’, ‘Satisfaction with current job’, ‘Independence at work’, ‘Opportunities for advancement’, and ‘Work schedule flexibility’ (Table 4).

**Table 4:** The effect of job characteristic on PES-NWI scale (Regression Model 1) and developed factors (Regression Model 2)

	REGRESION MODEL 1		REGRESION MODEL 2 (three factors, 19 statements)					
	PES-NWI (32 statements)		F1 - Equal involvement of nurses and participation in hospital affairs		F2 - Collegial nurse-physician relationships		F3 - Nurse manager ability, leadership and support of nurses	
	*(R*^*2*^ *= 0.480)*		*(R*^*2*^ *= 0.404)*		*(R*^*2*^ *= 0.268)*		*(R*^*2*^ *= 0.354)*	
*Variables*	*β*	*p*	*β*	*p*	*β*	*p*	*β*	*p*
Satisfaction – current job	0.183	**< 0.001**	0.065	0.093	-0.196	**< 0.001**	0.169	**< 0.001**
Satisfaction – career	0.010	0.753	0.030	0.363	0.010	0.788	0.048	0.155
Work schedule flexibility	0.021	0.545	-0.004	0.907	0.021	0.612	0.089	**0.020**
Opportunities for advancement	0.184	**< 0.001**	0.261	**< 0.001**	-0.119	**< 0.001**	0.101	**0.016**
Independence at work	0.068	0.067	0.058	0.140	-0.027	0.538	0.114	**0.005**
Professional status	0.150	**< 0.001**	0.056	0.170	-0.176	**< 0.001**	0.045	0.283
Wages	0.023	0.493	0.031	0.375	-0.045	0.245	-0.049	0.179
Educational opportunities	0.176	**< 0.001**	0.227	**< 0.001**	-0.084	**0.047**	0.210	**< 0.001**
Annual leave	0.005	0.894	0.003	0.935	0.032	0.467	0.011	0.785
Sick leave	0.044	0.275	0.000	0.995	-0.071	0.133	0.057	0.201
Study leave	0.91	**0.016**	0.133	**0.001**	-0.046	0.300	0.028	0.506
Education (HCA/BSN)	-0.083	**0.003**	-0.114	**< 0.001**	0.060	0.065	-0.024	0.437

Note. Note. R^2^ = Adjusted R-Squared, β = Standard regression coefficient, p = P-value. F1 – Equal involvement of nurses and participation in hospital affairs, F2 – Collegial nurse-physician relationships, F3 – Nurse manager ability, leadership and support of nurses

**Correlation is significant at the 0.01 level (2-tailed)

## Discussion

The study allows us to describe the context of the nursing work environment in surgical and internal medicine wards of participating Slovenian hospitals. The average achieved on the PES-NWI scale in our study was 2.65; this value is one of the lowest among previously conducted surveys. Original magnet and non-magnet hospitals received 2.95 and 2.65, respectively [25]. HCAs working on acute surgical and internal medicine wards were significantly less satisfied with their work environment and most of their work aspects compared to BSNs. The education and role of HCAs in Slovenia is comparable to the role of non-licensed practical nurses. Similarly, Phillips et al. [35] found this group of nursing care providers to have a lower self-assessment of the work environment and lower job satisfaction. Our study also showed HCAs to be less satisfied with their job, career, flexibility of work schedule, opportunities for development, autonomy at work, professional status, salary, educational opportunities, and leave. Not much research exists on the work environment of HCAs, with the bulk of research focusing on RNs [1–5, 11–14, 19–21, 36], including the RN4CAST study which does not include nursing staff with education lower than RNs [27]. In Slovenia, we decided to include HCAs in the study because this occupational group represents the majority of the bedside nursing workforce with a direct effect on the quality of care, although this effect is not measured. In Switzerland, the RN4CAST study conducted in 2020 included HCAs and their results showed HCAs to be more satisfied with their work environment compared to RNs and to have higher odds of also being satisfied with their job [37]; the exact opposite was shown by our results.

In their literature review, Swiger et al. [1] reported an average ratio range of PES-NWI scale from 2.30 to 3.07. The PES-NWI scale score is also validated by the answers to the question ‘How would you rate the working environment at your workplace in this hospital (resources, relationships, colleagues, support from supervisors?’ (2.70). Low score achieved through double self-assessment of the working environment can also be linked to the various aspects of work that received low scores in our study and the fact that nurses in Slovenia do not have opportunities for professional career development [38, 39], such as postgraduate specialisations, recognition of Master of Nursing degrees in clinical settings, and the creation of clinical nurse specialist and advance nurse practitioner posts [39]. Moreover, we are the European Union (EU) country with the lowest number of higher education nurses per capita according to the EU Directive [31, 40]. This situation is also reflected in our results on self-assessed aspects of work, where, in addition to dissatisfaction with wages, opportunities for advancement and education, as well as opportunities for professional development, also rated lower. These are predictors that significantly shape a nurse’s career and career choices [7, 21].

In the group of variables studied describing work characteristics, six variables proved to be significant and explained the self-assessed work environment in hospitals using the PES-NWI scale. The significant job characteristics were: ‘Opportunities for advancement’, ‘Satisfaction – current job’, ‘Educational opportunities’, ‘Professional status’, ‘Study leave’, and ‘Level of education’. The majority of these variables also explained the three dimensions of the PES-NWI scale in our study (regression model 2), additionally, the following two variables proved to be significant: ‘Work schedule flexibility’ and ‘Independence at work’. The dimensions were developed through a validation analysis of the globally known instrument PES-NWI scale [25] which was used for the first time in Slovenia.

Previous research confirms that the variables identified in the regression model of our study shape nurses’ work environment, but not to the extent that our study examines them using the PES-NWI scale as a dependent variable. In their literature review, Warshawsky et al. [41] summarise the predictors identified as explaining PES-NWI, but for each study they list no more than two to three variables that were significant in relation to self-assessed workplace characteristics and association with PES-NWI (nurse power, job satisfaction, organisational commitment, intention to resign, nurse satisfaction, clinical autonomy, and control over nursing practice). In their literature review of nine studies, Swiger et al. [1] report that findings for at least one of the nursing workplace characteristics were significant (with the composite score or subscale scores of PES-NWI). Some associations were significant at one level of analysis, but not at another. We believe that our study demonstrates the complexity of nurse workplace characteristics, and as such, it can meaningfully shape the activities of managers in Slovenian hospitals to improve nurses’ work environment.

### Implications for managers

Our study describes the work environment of two occupational groups and explains work environment characteristics which can be an invaluable tool for managers to accordingly improve work conditions of nursing employees in Slovenia. Considering the lack of BSNs in Slovenia, nursing care is being provided by a nursing team with very different educational qualifications and the effect of such care will have to be closely monitored. According to Aiken et al. [42], substituting RNs with nurses who have lower educational qualifications increases the risk for patient mortality.

The most important finding in our study is that satisfaction with wages, annual leave, and career choice has no effect on nursing employees’ self-assessment of the work environment. McHuge et al. [20] clearly state that wage is relevant for good nurse outcomes, but even more so are work environment and staffing. Our regression model and its dimensions reveal that career development opportunities and educational opportunities are crucial for the respondents, which is a response to their career development being hindered. These two variables explained the PES-NWI scale and its three dimensions to the greatest extent. Next came job satisfaction which explained two of the three work environment dimensions. Lu et al. [43] found that for hospital nurses, job satisfaction was closely related to work environment, empowerment culture, organisational and professional commitment, stress caused by the job, patient satisfaction, and patient-nurse-ratios, among others. In addition to the two established predictors (career development opportunities and educational opportunities), job satisfaction as a predictor affecting nurses’ work environment is an important finding of our study.

Collegial relationships may foster positive environments that make nurses more satisfied [44]. We can improve equal involvement and development of nurses and their participation in hospital management by improving the actual relationship between nurses and physicians and nurses and leaders. The same effect was observed on nurse manager ability, leadership and support of nurses. The relationship with physicians and their behaviour to nurses is crucial, as they influence the professional status of nurses, create their educational opportunities, and foster the development of nursing as a career and profession, which also prove to be important predictors in our study and others [1, 2, 4, 5, 21, 22] edit brackets. Respondents’ performance was below the acceptable score at the level of equal involvement when partnering with physicians. An ineffective or non-existent collaborative interaction leads to decreased empowerment, increased burnout, decreased job satisfaction, and increased turnover among nurses [2, 4, 15, 18].

It is clear that nursing management at different hospital levels must create support for professional practice to ensure workforce stability and provide optimal care, as was previously recognised by other studies which used the PES-NWI scale [45]. It is necessary to improve the nursing work environment; to support and encourage nurses’ continuing education, master’s, postgraduate education, and professional development; and to provide an environment for solidarity among colleagues [46]. Creating an optimal work environment for nurses is an important task for managers and leaders. Eva et al. [5] described interventions to improve the work environment as effective means of increasing job satisfaction. Nurses are encouraged to stay at their workplace by three crucial factors: effective leadership, empowerment possibilities, and professional development opportunities [4]. Just as importantly, the national healthcare policy must view nurses as equal healthcare professionals and nursing as a profession and a science.

We recognised that nursing practice environments are multifaceted and sensitive to context; some work environment characteristics may have more consistent effects across levels of analysis and context than others [44]. Fostering nurse empowerment, engagement, and good interpersonal relationships at work is essential for a positive work environment and high-quality patient care [2].

### Validation of PES-NWI scale and other limitations

The PES-NWI scale as a whole achieved very good reliability in our research [1, 30, 41], but in the context of construct validity we had to eliminate 13 items with low communalities. Across studies, researchers identified inconsistencies in the PES-NWI factor structure—some results corroborated the original factor structure, others adjusted the factor structure, altered items, and/or deleted items [1, 25, 41, 47, 48]. Our study results did not corroborate the original factor structure, we identified two factors from the original PES-NWI and developed one new factor which merged some items from two subscales developed by Lake [25]. Almeida et al.’s [49] final study results for the PES-NWI matched three domains of the original version, five items were excluded and belong to the Nursing Foundations for Quality of Care domain in the original version of Lake [25]. The partial alignment with the original PES-NWI scale has also been noted by other authors [48, 50–52]. We developed one new construct: ‘Equal development of nurses and their participation in hospital affairs’ (F1) which included also three statements from the subscale ‘Nursing foundations for quality of care’ with content staff development, philosophy of nursing pervading the patient care environment, and an active quality assurance program. The final three-factor solution had very good reliability and construct validity, but with 19 statements, two constructs by Lake [25] were not measured: ‘Staffing and resource adequacy’ and ‘Nursing foundation for quality of care’. In previous studies, some similar variations in construct validity have been observed. These can be due to differences in sample size, culture, healthcare systems, and cross-cultural differences reflected in the functioning of the healthcare system and management among countries as these may influence nurses’ responses [49, 53] and the decision about factorial weights. We used weights over 0.400 like some other authors [54, 55], while some other studies used weights over 0.300 [30]. We should understand this also as a signal that the items measuring particular dimensions of the nursing work environment may vary in different countries [53].

In analysing the data, we questioned also the appropriateness of measuring features on PES-NWI in four response categories in different languages. Taherdoos [56] describes attitude scales as any of a variety of scales that measure an individual’s predisposition toward any person, object or other phenomenon. Reliability is increased with increasing the number of response options. By increasing the numbers of scale points, validity will increase [56]. We believe that a minimum five-point scale should be used for the PES-NWI scale: 1 – Strongly Disagree, 2 – Disagree, 3 – Neither Agree nor Disagree, 4 – Agree, 5 – Strongly Agree.

The results are limited to the opinions of participating nurses. A higher response rate would be desired. The results of this study are limited to the answers provided by nurses working in the selected special fields and cannot be generalized to all hospital wards in Slovenia. A small part of the data (one hospital) was collected after the first wave of the epidemic, while all other data were collected before the start of the epidemic. We did not study the effect of the epidemic on the outcome as we would also need to capture pre-epidemic data for this hospital, but French et al. [57] showed that RNs in hospitals and nursing homes reported poor work conditions, high burnout, and poor patient safety and care quality before COVID-19 pandemic. It is difficult to be sure whether the situation has changed during and after the epidemic.

It is possible that the respondents were overly positive or negative towards their work environment, so caution should be applied in generalizing the findings. Next, caution should be used when generalizing and interpreting results in the regression model, as perceptions of the studied variables can vary from person to person and because cross-sectional research does not enable causal predictors to be determined, only the identification of predictors. Longitudinal and experimental research designs are more appropriate for predicting causal predictors. Finally, the accuracy of self-report survey techniques may be limited.

### Future research

The original PES-NWI scale was used in our study, just as by the RN4CAST consortium. Recommendations for PES-NWI scale include reducing scale length, employing consistent scoring methods, considering the impact of various modifications based on cultural and clinical setting nuances, and using the measure in longitudinal and intervention research designs [1, 41]. The instrument was used on a group of nurses working in internal medicine and surgery. More studies of PES-NWI scale in Slovenia could provide a different predictor structure to obtain an adequate PES-NWI model to apply across the Slovenian nursing context. The staffing and quality scales need to be re-analysed as they have proven to be less reliable and do not form a useful substantive construct. Future research of the nursing work environment should include all providers of direct nursing care, also those with educational qualifications lower than RNs.

## Conclusions

The study points to a low level of self-assessment of the work environment. Eight characteristics of the work environment were identified as possible improvements that are of great importance for nursing management, hospital management and policy makers in the development of nurses in Slovenia. A key finding of our study are the two variables which had the greatest effect on improving the work environment in nursing: career development opportunities and educational opportunities. Attention should be paid to recognising nurses as equals in the healthcare workforce who need to be empowered to develop the profession and have career development opportunities. Inter-professional relations and the equal involvement of nurses in hospital affairs are crucial. National healthcare policy must view nurses as equal healthcare professionals and nursing as a profession and a science. More longitudinal and interventional studies are needed to better understand nurses’ work environment.

## Data Availability

The data sets used and/or analysed during the current study are available from the first author of this article (Brigita Skela-Savič, bskelasavic@fzab.si). The datasets for this article are also publicly available on request at the RN4CAST consortium EU, Leuven Institute for Healthcare Policy, Department of Public Health & Primary Care, Katholieke Universiteit Leuven (Walter Sermeus, walter.sermeus@kuleuven.be).

## List of abbreviations

COVID: Corona Virus Disease
CAF: Confirmatory Factor Analysis
EU: European Union
HCA: Health Care Assistant
PAF: Principal Axis Factor Analysis
PCA: Principal Component Analysis
PES-NWI: The Practice Environment Scale of the Nursing Work Index
RN4CAST: Nurse Forecasting in Europe
